# Lipid Metabolism Traits Mediate the Effect of Psoriasis on Myocardial Infarction Risk: A Two-Step Mendelian Randomization Study

**DOI:** 10.3390/metabo13090976

**Published:** 2023-08-28

**Authors:** Yang Ding, Shengyi Yang, Mengjiao He, Shasha Fan, Xiaohua Tao, Wei Lu

**Affiliations:** 1 Center for Plastic & Reconstructive Surgery, Department of Dermatology, Zhejiang Provincial People’s Hospital, Affiliated People’s Hospital of Hangzhou Medical College, Hangzhou 310014, China; dingyang@hmc.edu.cn (Y.D.); fanshasha@hmc.edu.cn (S.F.); 2 Department of Infection Control, Second Afliated Hospital of Zhejiang University School of Medicine, Hangzhou 310009, China; 2523073@zju.edu.cn; 3 Departments of Environmental Health, School of Public Health, Hangzhou Normal University, Hangzhou 310009, China; dypp2nn@163.com

**Keywords:** psoriasis, lipid metabolism traits, myocardial infarction, mendelian randomization, mediation

## Abstract

Mendelian randomization (MR) analysis was performed to explore the effect of psoriasis on lipid metabolism traits and myocardial infarction (MI) risk and to analyze the proportion of the mediatory effect of lipid metabolism traits. Publicly accessible summary-level data for psoriasis, lipid metabolism traits, and MI were provided by the genome-wide association studies (GWASs) of the FinnGen Biobank, UK Biobank, and CARDIoGRAMplusC4D, respectively. A two-sample MR was carried out to evaluate the association of psoriasis with lipid metabolism traits and MI. Furthermore, the current research focused on determining if the impact of psoriasis on MI is mediated by lipid metabolism traits. The outcomes of the random effect inverse-variance-weighted (IVW) technique indicated a substantial link between genetically predicted psoriasis and a higher risk of low-density lipoprotein (LDL) cholesterol (OR: 1.006, 95% CI: 1.005–1.007, *p* = 0.024), apolipoprotein B (OR: 1.018, 95% CI: 1.010–1.026, *p* = 0.015), lipoprotein A (OR: 1.006, 95% CI: 1.002–1.010, *p* = 0.039), and MI (OR: 1.066, 95% CI: 1.014–1.121, *p* = 0.012). The percentages of the mediatory effect of LDL cholesterol, apolipoprotein B, and lipoprotein A under psoriasis conditions on MI risk was 7.4%, 10.2%, and 4.1%, respectively. Psoriasis was causally linked to an elevated risk of lipid metabolism levels and MI. This study further demonstrated that LDL cholesterol, apolipoprotein B, and lipoprotein A mediated the effect of psoriasis on MI risk. And timely lipid-lowering treatment should be given to MI patients.

## 1. Introduction

Psoriasis, a prevalent and chronic papulosquamous skin disease, imposes a significant societal burden [1]. Psoriasis affects around 125 million individuals globally and exhibits varying prevalence rates, ranging from 0.1% in East Asia to 1.5% in Western Europe, with the highest rates recorded in high-income countries [2,3]. Individuals with psoriasis are more likely to develop other chronic health diseases, such as depression, psoriatic arthritis, inflammatory bowel disease, and cardiometabolic syndrome [4].

The relationship between psoriasis and myocardial infarction (MI) has been controversial in epidemiologic studies [5]. Gelfand et al. found a substantially elevated adjusted relative risk of developing MI among individuals with severe psoriasis after adjusting for major cardiovascular risk factors [6]. Several studies have confirmed the independent association of psoriasis with an escalated risk of developing MI [7,8,9]. Nevertheless, results from a large population-based Dutch cohort study indicated that psoriasis might not be an independent risk factor for MI [10]. The lasted new meta-analysis of 31 cohort studies revealed a 17% higher risk of MI in individuals with psoriasis in comparison to that in healthy individuals, with pooled ORs of 1.11 to 1.24 [11]. Furthermore, another experimental study by Siddabasave Gowda B Gowda found a significantly decreased lipidome in human ischemic LV and differential lipid metabolites in the transition of acute-to-chronic HF with inter-organ communication [12]. Other genome-wide association studies have identified a genetic locus at human chromosome 8q24 as having minor alleles associated with lower levels of plasma lipids, as well as decreased risk for myocardial infarction. Other studies have provided functional evidence for a novel gene regulating hepatic lipogenesis and VLDL production in mice that influences plasma lipids and the risk of myocardial infarction in humans [13]. Notably, most of these results come from observational studies, which are insufficient for conclusions due to limitations in design, sample sizes, and confounders [14].

Furthermore, the mechanisms underlying the impact of psoriasis on MI risk are not fully comprehended. In a previous report, psoriasis was linked to abnormalities in lipid traits (triglycerides, LDL-C, HDL-C, and total cholesterol), with ORs ranging from 1.04 to 5.55 [15]. Meanwhile, clinical trials have demonstrated that a reduction in blood lipid levels reduces MI events [16]. Thus, one of the mechanisms may be mediated by lipid metabolism traits (a cardiovascular risk factor) that increase MI risk. However, the degree to which these lipid metabolism characteristics account for the total impact of psoriasis on MI remains unexplored. Mendelian randomization (MR) analysis overcomes the constraints of traditional methods by employing genetic markers as instrumental variables (IVs). This approach helps to determine the correlation between predicted risk factors and disease [17,18]. These advantages also extend to mediation analysis, whereas methods for mediation analysis that do not employ instrumental variables experience multiple methodological challenges [19]. In the present research, MR was performed to examine the impact of psoriasis on lipid metabolism traits and MI risk and also to determine the proportion of the mediatory effect of lipid metabolism traits.

## 2. Materials and Methods

### 2.1. Study Design

**Figure 1:**
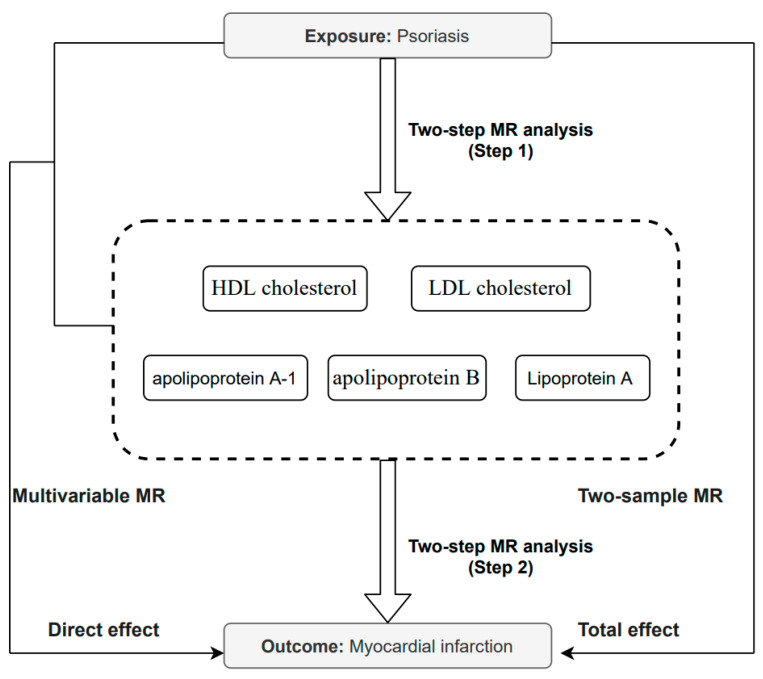
Overview of the MR design of the present study. HDL cholesterol, high-density lipoprotein cholesterol; LDL cholesterol, low-density lipoprotein cholesterol.

The current research involved a two-sample MR analysis to assess the association of psoriasis with MI, referred to as the total effect. To further explore if lipid metabolism traits act as mediators of the effects of psoriasis on disease, another two-step MR was conducted. Initially, the link between psoriasis and lipid traits was assessed. Subsequently, the effect of lipid traits on MI risk was analyzed. Finally, multivariable MR was carried out to identify the direct effect of psoriasis on MI, which was subtracted from the total effect to obtain an estimate of the indirect effects. The summary of the current research design is shown in Figure 1.

### 2.2. Data Retrieval or MR

The current research acquired comprehensive data on the correlation between the single nucleotide polymorphism (SNP) and phenotype from various genome-wide association studies (GWASs). Publicly accessible summary-level data for psoriasis were acquired from the GWAS using data from FinnGen Biobank. The FinnGen study is a unique study that integrates genomic data with digital health care records of more than 500,000 Finnish biobank participants [20].

Summary statistic data for lipid traits, including LDL-C, HDL-C, apolipoprotein B, apolipoprotein A-1, and lipoprotein A, were retrieved from a genome-wide association meta-analysis of 35 biomarkers. These data are deposited in the UK Biobank (UKB) and consist of information from 304,818 participants of European descent [21]. The UKB data were derived from a prospective cohort investigation that included over 500,000 men and women aged between 40 and 69 at the start of the study between 2006 and 2010 [22].

Summary statistic data for MI were obtained from a genome-wide association meta-analysis of 185,000 coronary artery disease (CAD) cases and controls. This analysis assessed 6.7 million common variants with a minor allele frequency (MAF) > 0.05 and 2.7 million low-frequency (0.005 < MAF < 0.05) variants [23]. The specifics of the data utilized in this research are shown in Table 1.

### 2.3. Selection of Genetic IVs and Data Harmonization

The genetic variants utilized as IVs for psoriasis had a genome-wide significance (*p* < 5 × 10^−8^) and were distinct from the variants for lipid traits [24]. Similarly, genetic variants for lipid traits were not shared with those for psoriasis. For all experiments, the independent distribution of IVs was achieved by pruning SNPs within a 10,000 kb window based on a threshold of *r*^2^ < 0.001 [25]. SNPs linked to body mass index and hypertension were identified as pleiotropic IVs. They were obtained by exploring the GWAS Catalog (https://www.ebi.ac.uk/gwas/ (accessed on 1 June 2022)) and PhenoScanner (http://www.phenoscanner.medschl.cam.ac.uk/ (accessed on 1 June 2022)) using MI as an outcome to help eradicate any potential pleiotropic effects [26]. Following this, SNPs linked to exposure were acquired from the outcome datasets. Furthermore, SNP harmonization was carried out to correct for the allele orientation. The specifics regarding the selection of variables are shown in Supplementary Materials Tables S1–S11.

### 2.4. Instrument Strength

The F-statistic value for each instrument-exposure effect was above 10, suggesting a low risk of weak instrument bias. The F-statistic was computed using the following formula: F = [(N − K − 1)/K] × [R^2^/(1 − R^2^)], where R^2^ indicates the proportion of variance in instruments according to the formula R^2^ = 2 × (1 − MAF) × MAF × (Beta/stand error)^2^. The F-statistic value for each instrument-exposure association ranged from 15.996 to 64.356, revealing less likelihood of a weak IV bias in the final results, as detailed in Supplementary Materials Tables S1–S11.

### 2.5. Mediation Analysis and the Proportion of the Mediation Effect

The total effect of exposure on an outcome can be classified into two components: indirect and direct effects [19]. The immediate effect of psoriasis on MI risk was achieved after adjusting for LDL-C, apolipoprotein B, and lipoprotein A through multivariable MR. The research involved performing a multivariable MR of the effect of psoriasis on MI by considering all mediators as a comparison. The multivariable MR used a set of genetic proxies that were at least associated with psoriasis, LDL-C, apo B, or Lpa to predict each of the variables in the model [19,27]. These predicted values were used to estimate the effect of psoriasis on MI after adjusting for mediators in a multivariable regression analysis. The indirect effect of each lipid trait was derived by utilizing the product method, which involved multiplying the effect of psoriasis on lipid traits by the effect of lipid traits on MI [28]. Additionally, a multivariable MR was conducted to analyze the effect of each mediator on MI after adjusting for psoriasis.

The potential mediators were determined by estimating the variations in the total effect of the genetically determined psoriasis on the risk of MI. The following formula was applied to estimate the proportion of the mediation effect [29]:

$$E\left(\%\right)=\frac{\sum_{k=1}^{k}\beta 1\times \beta 2k}{\sum_{k=1}^{k}\beta 3+\beta 1\times \beta 2k}$$

where *β*1 denotes the MR effect of psoriasis on a mediator, *β*2 denotes the MR effect of mediator *k* on MI after adjusting for genetically determined psoriasis, and *β*3 is the MR effect of psoriasis on MI after adjusting for a genetically determined potential mediator.

### 2.6. Mendelian Randomization Estimates

The primary analysis utilized the inverse-variance-weighted (IVW) technique to pool Wald ratio estimates of the causal impacts of various SNPs [30]. The application of the IVW technique presupposes that all SNPs are valid IVs; thus, this method can help achieve accurate estimation results [31]. However, given that the IVW method can exhibit bias even if a single genetic variant is invalid (i.e., if only a single variant exhibits horizontal pleiotropic effects) [32], complementary analyses were carried out by employing the MR-egger method [33], weighed median method [32], and maximum likelihood method [30]. MR-egger approach comprises a weighted linear regression of the gene–outcome coefficients on the gene–exposure coefficients [33]. The weighted median method offers a consistent estimate of the causal effect, provided that at least half of the SNPs are valid IVs [32]. Furthermore, the MR-robust adjusted profile score (MR-RAPS), utilizing the “Huber” loss function, was employed to account for the random-effects distribution of pleiotropic effects of the genetic variants in the model [34].

### 2.7. Heterogeneity and Pleiotropy Analysis

Cochran’s *Q* statistic was utilized to calculate heterogeneity. The final MR results were analyzed utilizing a multiplicative random-effects model of IVW if the *p* value of Cochran’s *Q* test was below 0.05; otherwise, a fixed-effects model was employed [35]. The MR-egger intercept was determined to test for bias attributed to directional pleiotropy, where the average of the direct effects of the tested genetic variants on outcome was non-zero, and the intercept demonstrated the average pleiotropic impact across the genetic variants [33]. Finally, leave-one-out sensitivity analyses were performed, whereby a single SNP was removed at a time to examine if a single SNP was responsible for the causal association.

Analyses were carried out by utilizing R software version 4.0.5 using “Two-Sample-MR” and “MR-RAPS” packages. *p* < 0.05 denoted the significance level.

## 3. Results

### 3.1. Effect of Psoriasis on Lipid Metabolism Traits

**Figure 2:**
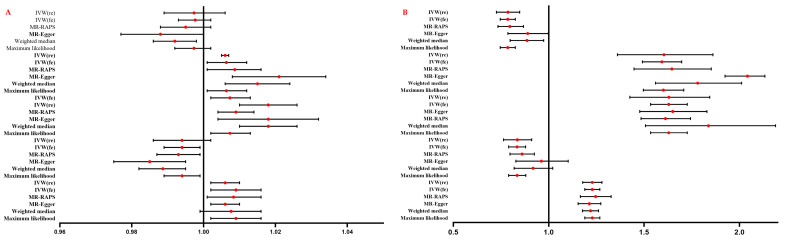
(**A**). Effect of psoriasis on lipid metabolism traits; (**B**) effect of lipid metabolism traits on MI. HDL-C, high-density lipoprotein cholesterol; LDL-C, low-density lipoprotein cholesterol; Apo B, apolipoprotein B; Apo A1, apolipoprotein A1; Lp A, Lipoprotein A; IVW(re), inverse variance weighted with random effect; IVW(fe), inverse variance weighted with fixed effect; MR-RAPS, MR-Robust adjusted profile score.

The findings of the random-effect IVW method showed a substantial link between genetically predicted psoriasis and the enhanced risk of LDL cholesterol (OR: 1.006, 95% CI: 1.005–1.007, *p* = 0.024), apolipoprotein B (OR: 1.018, 95% CI: 1.010–1.026, *p* = 0.015), and lipoprotein A (OR: 1.006, 95% CI: 1.002–1.010, *p* = 0.039, Figure 2A, Supplementary Table S12). The causal estimates remained consistent across all applied MR methods, with the exception of the effect on lipoprotein A determined with the weighted median method (Figure 2A). However, no causal effect of psoriasis on HDL cholesterol (OR: 0.997, 95% CI: 0.989–1.006, *p* = 0.534) and apolipoprotein A1 abnormalities (OR: 0.994, 95% CI: 0.986–1.002, *p* = 0.142) was determined using the random-effect IVW method (Figure 2A, Supplementary Table S12). The results of heterogeneity and pleiotropy analysis are presented in Table 2. The F-statistic value for the instrument–psoriasis link was 15.996 in the HDL, LDL, apolipoprotein A1, and apolipoprotein B model and 16.533 in the lipoprotein A model (Supplementary Tables S1–S5). 

### 3.2. Effect of Lipid Metabolism Traits on MI

Results of the random-effect IVW method highlighted a substantial association of genetically predicted LDL cholesterol (OR: 1.591, 95% CI: 1.360–1.861, *p* < 0.001), apolipoprotein B (OR: 1.621, 95% CI: 1.425–1.844, *p* < 0.001), and lipoprotein A (OR: 1.228, 95% CI: 1.178–1.279, *p* < 0.001) with an increased MI risk, whereas HDL cholesterol (OR: 0.785, 95% CI: 0.726–0.848, *p* < 0.001) and apolipoprotein A1 (OR: 0.834, 95% CI: 0.763–0.911, *p* < 0.001) exhibited a substantial correlation with a reduced risk of MI (Figure 2B, Supplementary Table S13). The results of other implemented MR methods are presented in Figure 2B, and the results of heterogeneity and the pleiotropy analysis are presented in Table 2. The F-statistic values for each instrument–lipid association were 49.720, 50.535, 47.418, 49.259, and 64.356 for HDL cholesterol, LDL cholesterol, apolipoprotein B, apolipoprotein A1, and lipoprotein A (Supplementary Tables S7–S11).

### 3.3. Effect of Psoriasis on MI

**Figure 3:**
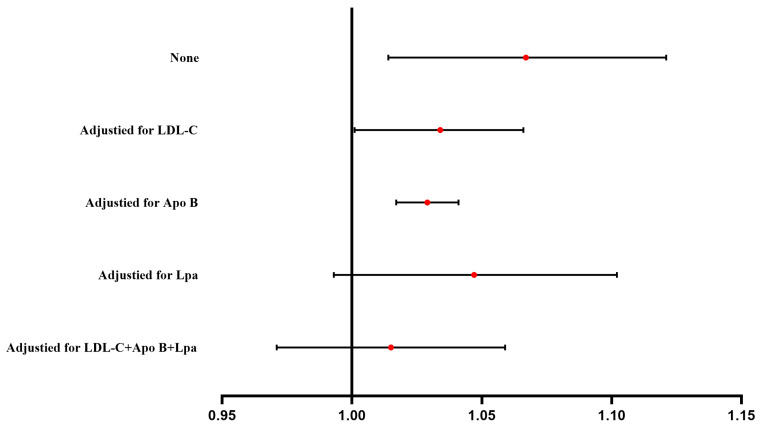
The effect of genetically predicted psoriasis on myocardial infarction risk after adjusting for genetically predicted LDL-C, Apo B, and Lpa, either separately or in the same model. LDL-C, low-density lipoprotein cholesterol; Apo B, apolipoprotein B; Lpa, Lipoprotein A.

The findings obtained with the fixed-effect IVW method showed a substantial link between genetically predicted psoriasis and an elevated MI risk (OR: 1.066, 95% CI: 1.014–1.121, *p* = 0.012, Figure 3, Supplementary Table S12). The results were consistent across all applied MR methods, with the exception of the effect on MI determined with weighted median and MR egger techniques (Supplementary Table S12). Moreover, no heterogeneity and pleiotropy were detected. The F-statistic value for the instrument–psoriasis association was 16.995 (Supplementary Table S6).

### 3.4. Proportion of the Mediatory Effect of LDL Cholesterol, Apolipoprotein B, and Lipoprotein A

According to the above analysis outcomes, LDL cholesterol, apolipoprotein B, and lipoprotein A mediate the effect of psoriasis on the MI risk. In the multivariable MR of psoriasis—LDL cholesterol—MI, the direct effect of psoriasis on MI was reduced to an OR of 1.035 (95% CI: 1.001, 1.066, *p* = 0.038, Table 3, Figure 3). Furthermore, the direct effect of psoriasis on MI was also attenuated in the multivariable psoriasis–apolipoprotein B–MI MR (OR: 1.029, 95% CI: 1.017–1.041, *p* = 0.046, Table 3, Figure 3). Following an adjustment for lipoprotein A, no causal effect of psoriasis on MI was detected (Table 3, Figure 3). Furthermore, no causal effect of psoriasis on MI was observed when the three lipid metabolism traits were added to the same model (Table 3, Figure 3). The proportions of the mediatory effect of LDL cholesterol, apolipoprotein B, and lipoprotein A were 7.4%, 10.2%, and 4.1%, respectively. When all three lipid traits (LDL cholesterol, apolipoprotein B, and lipoprotein A) were incorporated into the same model, the combined percentage was 11.6%. 

## 4. Discussion

According to the current literature, this is the first study investigating how lipid metabolism traits mediate the causal path between psoriasis and MI. This research discovered that genetically determined psoriasis exhibited a possible correlation between elevated levels of lipid metabolism and MI risk. Moreover, it was observed that LDL cholesterol, apolipoprotein B, and lipoprotein A mediated the psoriasis effect on MI risk.

Recent observational studies have explored the causal association between psoriasis and MI. A cross-sectional patient–population study of 113,065 patients in Japan reported that psoriasis vulgaris exhibited a substantial link to an 87% elevated MI risk [8]. Following an adjustment for age and gender, psoriasis vulgaris was found to have an independent association with MI (adjusted OR = 1.49) in adults. However, the population involved in this study consisted of patients from a Japanese hospital with no information available about the severity and treatment of psoriasis vulgaris, which could have caused selection bias. A retrospective cohort study of the American population also found that mild and severe psoriasis patients exhibited a remarkable link to 31% and 28% increased risks of MI compared to those in matched control patients, respectively [36]. While psoriasis has been associated with MI, the results from various epidemiological studies remain inconsistent. The discrepancies in the results could be attributed to varying factors, such as the severity and duration of psoriasis, and the presence or absence of arthritis, all of which can influence the impact of psoriasis on MI [37,38]. Another cross-sectional and cohort study that used various large-scale epidemiological and genetic datasets also indicated that psoriasis is an independent yet modest risk factor for MI with a “dose-response” association, as severe psoriasis was linked to an elevated MI risk [39].

Notably, the above outcomes are primarily derived from observational studies that are insufficient to make conclusions due to limitations in the design, sample sizes, and confounders. The MR analysis in the current study that utilized genetic variants as IVs provided evidence of the effect of psoriasis on MI. The confounder effect was overcome by extracting the confounder-related SNPs when the IVs were selected. One possible explanation for the link between psoriasis and MI could be that these entities share common genetics. However, a comprehensive GWAS indicated that with the exception of two psoriasis risk loci that had a minor influence on the CAD risk (OR < 1.2), no other recognized psoriasis risk polymorphisms displayed a strong correlation with CAD [39]. Since the genetic overlap does not sufficiently elucidate the heightened risk of MI in psoriasis patients, further research is needed to investigate the cardiovascular risk factors, which might serve as the mediatory factors of the psoriasis–MI pathway. Furthermore, systemic inflammation could function synergistically with metabolic abnormalities to enhance the cardiovascular risk in these patients [5,40,41,42].

Lipid metabolism abnormalities, closely related to psoriasis and MI, might be an intermediate factor along the pathway from psoriasis to MI. Two meta-analyses highlighted that LDL cholesterol, lipoprotein A, and apolipoprotein B levels were considerably higher in individuals with psoriasis than in controls [15,43]. LDL particles undergo oxidative modification and produce oxidized LDL (ox-LDL). This ox-LDL can enter macrophages, inducing their transformation into foam cells, which contribute to the development of atherosclerotic plaques [40]. As a composite measure of all apolipoprotein B-containing lipoproteins, apolipoprotein B was also associated with developing MI risk [44]. The effect of psoriasis on MI was decreased when the current investigation involved an adjustment for lipid metabolism traits in the multivariable MR model, indicating that LDL cholesterol, apolipoprotein B, and lipoprotein A were the intermediate factors. Lipoprotein A is an LDL-like lipoprotein with apolipoprotein covalently linked to apolipoprotein B via a disulfide bond [45]. It has been proven that lipoprotein A plays crucial roles in atherosclerosis, such as foam cell formation, smooth muscle cell proliferation, and plaque inflammation and instability [46].

The present study has several notable strengths. First, publicly accessible summary-level data for psoriasis, lipid metabolism traits, and MI were drawn from several large-scale consortium data of European populations, enhancing the statistical power of the data. Second, the current research employed a range of techniques for sensitivity analysis, especially excluding SNPs associated with potential confounding factors, to improve estimation reliability. Third, the genetic variants utilized as the IVs were located on distinct chromosomes, thus minimizing the impact of potential gene–gene interactions on the estimated value. Fourth, using mediation, MR reduced the bias due to confounding among exposure factors, mediators, outcomes, and measurement error. Finally, multivariable MR could accommodate the combined effects of numerous mediators, even when bidirectional relationships were present [19].

Nevertheless, the study also has several limitations. First, exposure-associated SNPs explained a relatively small proportion of the variation, thus restricting the statistical power to identify a weak correlation between the genetically predicted exposures and outcomes. However, the F-statistic value exceeding 10 minimized any bias resulting from the use of a weak instrument in the analysis. Second, although the heterogeneity was presented in this study, a GWAS with different ages, sexes, and health statuses could not be obtained to explore the resources of heterogeneity. Third, the success of mediation analysis crucially depended on the correct pre-established formulation of the causal relationships of the exposures, as the mediatory and confounding effects could not be statistically distinguished [47]. Furthermore, the current research did not consider the bidirectional effect between psoriasis and lipid metabolism. Finally, the data used in the study were from the GWAS of MI involving a population of South Asian descent, which could potentially result in bias from the non-European population.

## 5. Conclusions

This MR study indicated a causal association between genetically determined psoriasis and an elevated risk of lipid metabolism levels and MI, with evidence that the LDL cholesterol, apolipoprotein B, and lipoprotein A mediated the effect of psoriasis on the MI risk. Therefore, more attention should be paid to the lipid metabolism levels of patients with psoriasis for the primary prevention of MI. And timely lipid-lowering treatment should be given to MI patients. 

## Figures and Tables

**Table 1:** Details of the GWAS included in Mendelian randomization analyses.

Trait	Consortium	Ethnicity	Sample Size (Case and Control)
Psoriasis (*n*, %)	FinnGen Biobank	European	216,752 (4510 and 212,242)
HDL cholesterol (mmol/L)	UK Biobank	European	403,943
LDL cholesterol (mmol/L)	UK Biobank	European	440,546
apolipoprotein B (mmol/L)	UK Biobank	European	439,214
apolipoprotein A-1 (mmol/L)	UK Biobank	European	393,193
Lipoprotein A (mmol/L)	UK Biobank	European	5732
Myocardial infarction (*n*, %)	CARDIoGRAMplusC4D	European	171,875 (43,676 and 128,199)

HDL cholesterol, high-density lipoprotein cholesterol; LDL cholesterol, low-density lipoprotein cholesterol; CARDIoGRAMplusC4D, Coronary ARtery DIsease Genome wide Replication and Meta-analysis plus Coronary Artery Disease Genetics Consortium.

**Table 2:** Heterogeneity and pleiotropy analysis.

Exposure	Outcome	No. of SNPs	R2 (%)	Heterogeneity				Pleiotropy	
				Method	Cochran’s Q	I2 (%)	*p*-Value	Egger-Intercept (95% CI)	*p*-Value
Psoriasis	HDL cholesterol	12	0.0886	IVW	35.183	57.34	<0.001	−0.004 (−0.006, 0.002)	0.492
Psoriasis	LDL cholesterol	12	0.0886	IVW	46.187	61.24	0.004	0.002 (−0.004, 0.008)	0.396
Psoriasis	apolipoprotein B	12	0.0886	IVW	11.000	65.49	<0.001	−0.004 (−0.008, 0.000)	0.077
Psoriasis	apolipoprotein A1	12	0.089	IVW	28.134	43.48	0.003	−0.003 (−0.007, 0.001)	0.355
Psoriasis	Lipoprotein A	11	0.084	IVW	14.253	35.36	0.162	0.001 (−0.003, 0.005)	0.655
HDL cholesterol	MI	317	3.903	IVW	753.222	37.62	<0.001	−0.004 (−0.008, 0.000)	0.066
LDL cholesterol	MI	146	1.752	IVW	847.515	82.12	<0.001	−0.001 (−0.009, 0.007)	0.677
apolipoprotein B	MI	178	1.871	IVW	825.557	79.57	<0.001	−0.005 (−0.010, 0.001)	0.103
apolipoprotein A1	MI	261	3.46	IVW	724.154	63.45	<0.001	−0.005 (−0.013, 0.003)	0.182
Lipoprotein A	MI	14	0.196	IVW	21.276	39.74	0.068	0.010 (−0.018, 0.032)	0.383
Psoriasis	MI	8	0.032	IVW	1.583	2.80	0.979	0.001 (−0.029, 0.031)	0.989

**Table 3:** Multivariate MR analysis of the direct effect of psoriasis on MI.

Exposure/Outcome	Adjustied Factors	Multivariate MR Analysis				
		nSNP	OR (95% CI)	*p*-Value	Mediation Effect	Mediation Effect (%)
Psoriasis/Myocardial infarction	None	8	1.066 (1.014, 1.121)	0.012	/	/
Psoriasis/Myocardial infarction	LDL cholesterol	162	1.035 (1.001, 1.066)	0.038	0.003 (0.002, 0.004)	7.4
Psoriasis/Myocardial infarction	apolipoprotein B	180	1.029 (1.017, 1.041)	0.046	0.004 (0.002, 0.003)	10.2
Psoriasis/Myocardial infarction	Lipoprotein A	26	1.046 (0.993, 1.102)	0.091	0.002 (0.001, 0.003)	4.1
Psoriasis/Myocardial infarction	LDL cholesterol apolipoprotein B Lipoprotein A	323	1.015 (0.971, 1.059)	0.399	0.009 (0.007, 0.010)	11.6

LDL cholesterol, low-density lipoprotein cholesterol; OR, odds ratio.

## Data Availability

The data presented in this study are available in supplementary.

## Supplementary Materials

The following supporting information can be downloaded at: https://www.mdpi.com/article/10.3390/metabo13090976/s1, Table S1. Harmonized dataset of Mendelian randomization for the effect of Psoriasis on HDL-C; Table S2. Harmonized dataset of Mendelian randomization for the effect of Psoriasis on LDL-C; Table S3. Harmonized dataset of Mendelian randomization for the effect of Psoriasis on Apo B; Table S4. Harmonized dataset of Mendelian randomization for the effect of Psoriasis on Apo A1; Table S5. Harmonized dataset of Mendelian randomization for the effect of Psoriasis on Lpa; Table S6. Harmonized dataset of Mendelian randomization for the effect of Psoriasis on MI; Table S7. Harmonized dataset of Mendelian randomization for the effect of HDL-C on MI; Table S8. Harmonized dataset of Mendelian randomization for the effect of LDL-C on MI; Table S9. Harmonized dataset of Mendelian randomization for the effect of Apo B on MI; Table S10. Harmonized dataset of Mendelian randomization for the effect of Apo A1 on MI; Table S11. Harmonized dataset of Mendelian randomization for the effect of Lpa on MI; Table S12. Effect of psoriasis on lipid metabolism traits; Table S13. Effect of lipid metabolism traits on MI.
